# In Vitro Assay of Translation Inhibition by Trichothecenes Using a Commercially Available System

**DOI:** 10.3390/toxins13100696

**Published:** 2021-10-01

**Authors:** Takahito Toyotome, Katsuhiko Kamei

**Affiliations:** 1 Department of Veterinary Medicine, Obihiro University of Agriculture and Veterinary Medicine, Obihiro 080-8555, Japan; 2 Diagnostic Center for Animal Health and Food Safety, Obihiro University of Agriculture and Veterinary Medicine, Obihiro 080-8555, Japan; 3 Medical Mycology Research Center, Chiba University, Chiba 260-8673, Japan; k.kamei@faculty.chiba-u.jp

**Keywords:** trichothecenes, in vitro assay, cell-free assay, protein synthesis inhibition

## Abstract

Trichothecenes are a family of major secondary metabolites produced by some common filamentous fungi, including plant pathogenic and entomopathogenic fungi. It may be considered difficult to conduct a comparison between the toxicities of trichothecenes with consideration of different conditions and cell lines. In the current study, we developed an in vitro assay based on a commercially available system to estimate the translation inhibition, that is, the main toxicity, of trichothecenes. The assay was applied to estimate the inhibition of protein synthesis by trichothecenes. Initially, we examined the assay using trichothecene dissolved in water followed by an assessment of trichothecene solutions dissolved in acetonitrile. The obtained data showed that the assay tolerated the small amount of acetonitrile. The assay examined in this study has the advantages of a short operation time (one day), ease of use, and data stability, as it is a non-cell-based assay whose components are commercially available. It is expected that this assay will contribute to the evaluation of the toxicity of a vast number of trichothecenes.

## 1. Introduction

Trichothecenes are a family of major secondary metabolites produced by some filamentous fungi, such as *Fusarium*, *Myrothecium*, *Trichothecium*, and *Stachybotrys*, and include more than 200 compounds, which have been classified into types A, B, C, and D. Their producers are various species, including plant pathogenic and entomopathogenic fungi [1,2,3]. In particular, trichothecenes produced by *Fusarium* spp. are strongly associated with human and animal health and food safety.

Trichothecenes are known as causative agents of severe food poisoning. T-2 and HT-2 toxins are important type B trichothecenes produced by *Fusarium* spp., such as *F. armeniacum* and *F. sporotrichioides*. A disease known as alimentary toxic aleukia (ATA) occurred in the 1930s and 1940s in Russia, whose occurrence was strongly associated with the consumption of overwintered grains infected with *F. sporotrichioides* and *F. poae*. Later, T-2 toxin was discovered and showed the induction of ATA-like symptoms [4]. The *F. graminearum* species complex is a major producer of DON and the major causative agent of a red mold disease, or “akakabi-byo”, in wheat. In Asia, red mold intoxications have been recorded as human poisoning episodes [5,6], and DON has been confirmed as the major contaminant in wheat related to at least one outbreak [7]. These toxins induce gastrointestinal disorders, including nausea, vomiting, and diarrhea in humans and animals. T-2 toxin induces leukopenia or aleukia as associated with ATA. 

Trichothecenes bind to and cleave ribosomal RNA [8,9,10], which leads to the inhibition of protein synthesis. The inhibition is the main effect of trichothecenes [11] and is thought to broadly affect intracellular processes, including signal transduction [12], DNA synthesis [13], nitric oxide (NO) release [14], caspase cleavage [9], and apoptosis induction [9], and it also leads to cell death [14,15] and various toxic effects, including immune responses [16,17]. 

Deepoxy-deoxynivalenol (DOM-1) is a 12,13-epoxy ring-opened form of DON, which is produced through microbial biotransformation by intestinal and ruminal microbes [12,18,19]. The epoxy group is essential for the toxicity of DON [20] and other trichothecenes [21,22]. 

The acute toxicity of trichothecenes has been assessed and compared by in vivo [23,24,25] and in vitro assays using cell lines [12,13,14]. However, it may be considered difficult to conduct a comparison between the toxicities of trichothecenes with consideration of different conditions, different cell lines, and different laboratory strains of the same cell lines. We expected that a rapid and simple in vitro evaluation would provide stable comparisons of toxicity among trichothecenes. Here, we used an in vitro assay to estimate the translation inhibition, that is the main toxicity, of trichothecenes. 

## 2. Results

### 2.1. DON, but Not DOM-1, Inhibits Protein Synthesis in a Cell-Free In Vitro System

**Figure 1:**
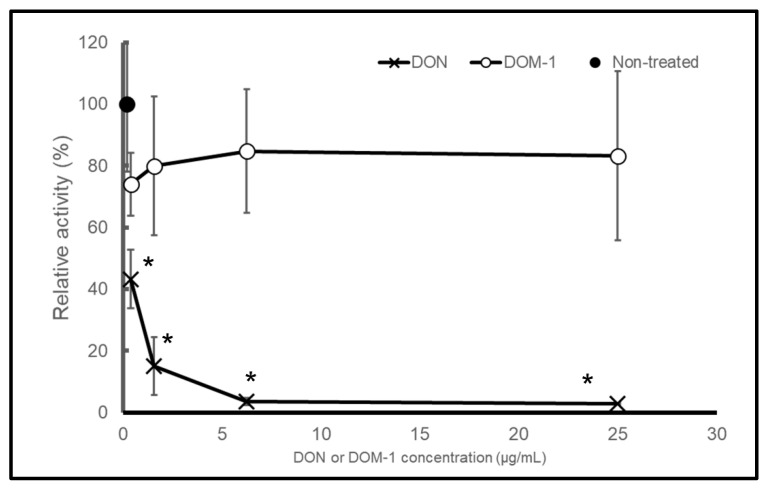
Comparisons of relative β-galactosidase activity after protein synthesis in the cell-free in vitro system treated with DON and DOM-1 for three hours. A filled circle (⦁) is a non-treated condition (water only). Cross marks (×) and open circles (∘) indicate the relative β-galactosidase activities of DON-treated and DOM-1-treated samples, respectively. These are relative values compared to the non-treated sample. Symbols represent the mean ± standard deviation. Asterisks (*) mean *p* < 0.01.

The Human Cell-Free Protein Expression System (TaKaRa Bio Inc., Shiga, Japan), an in vitro protein synthesis system, was used for transcription with RNA polymerase of T7 bacteriophage and translation with a cell lysate-derived human cell line in this study. The system contains a plasmid carrying a β-galactosidase gene as a positive control plasmid. The volume of the plasmid (0.3 µg/mL) was altered from 1 µL as indicated in the instructions to 0.5 µL, and 0.5 µL ultra-pure water was added to the reaction mixture. The β-galactosidase protein was produced in the modified reaction mixture. Next, instead of 0.5 µL of ultrapure water, a DON solution (0.39, 1.6, 6.3, and 25 µg/mL at final concentration) was added to the reaction mixture. As the concentration of DON increased, the amount of β-galactosidase produced decreased (Figure 1, cross marks). Next, a DOM-1 solution was added instead of the DON solution, and no reduction in β-galactosidase production was observed, even at 25 µg/mL of DOM-1 (Figure 1, open circles). These data indicate that the system can be applied to estimate the inhibition of protein synthesis by trichothecenes.

### 2.2. Acetonitrile as a Solvent Has No Preventative Effect on Protein Synthesis

**Figure 2:**
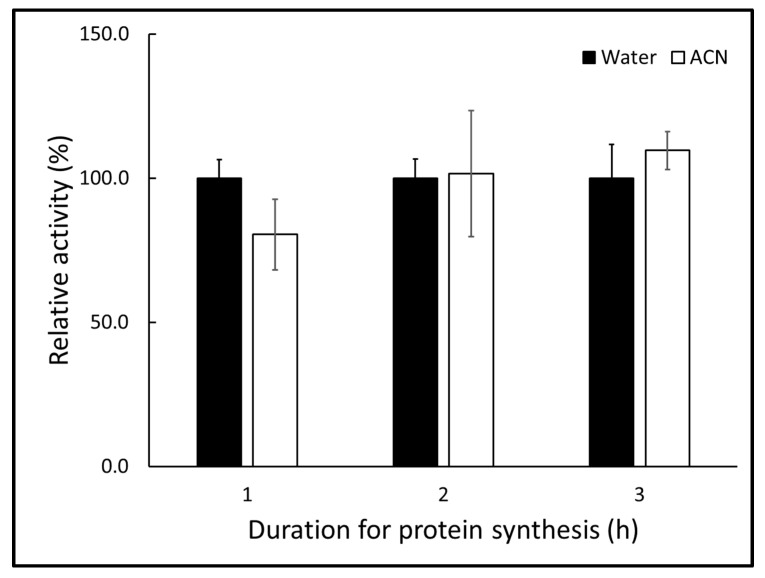
Comparisons of relative β-galactosidase activity after protein synthesis in the cell-free in vitro system with water (100%) or ACN added instead of trichothecene solution. Bars represent the mean ± standard deviation. There were no significant differences (*p* > 0.05) between the protein synthesis in experiments with water or ACN.

Some trichothecenes such as T-2 and HT-2 toxins are difficult to dissolve in water. Most trichothecene solutions are available as acetonitrile (ACN) solutions. To assay water-insoluble trichothecenes, we substitute ACN for water in the protein synthesis system. As shown in Figure 2, the β-galactosidase activities of ACN-containing samples were comparable to those of control samples with added water only. These data show that the assay tolerated the small amount of ACN.

### 2.3. T-2 and HT-2 Toxins Show Stronger Inhibition than that of DON

**Figure 3:**
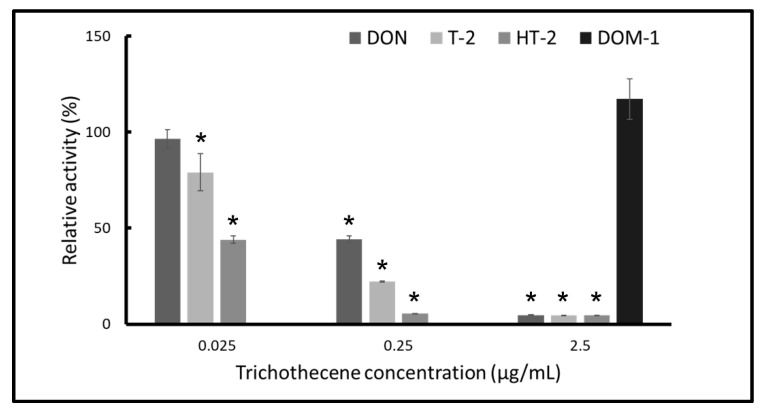
Comparisons of relative β-galactosidase activity after protein synthesis in the cell-free in vitro system treated with DON, T-2 toxin, and HT-2 toxin for one hour. Trichothecenes were dissolved in ACN. The relative activities are against the control samples with acetonitrile added. These values were compared to the control sample. Bars represent the mean values ± standard deviation. Asterisks (*) mean *p* < 0.01.

We examined the inhibition of the in vitro protein synthesis with three trichothecenes, namely, DON, T-2 toxin, and HT-2 toxin, as well as DOM-1 (Figure 3). Other than DOM-1, the tested trichothecenes inhibited proteins synthesis in a dose-dependent manner. In the assay, the inhibition potency of HT-2 toxin was the strongest, and the inhibition potency of DON was the weakest.

### 2.4. Effect on the Transcription Step in the Presence of DON

**Figure 4:**
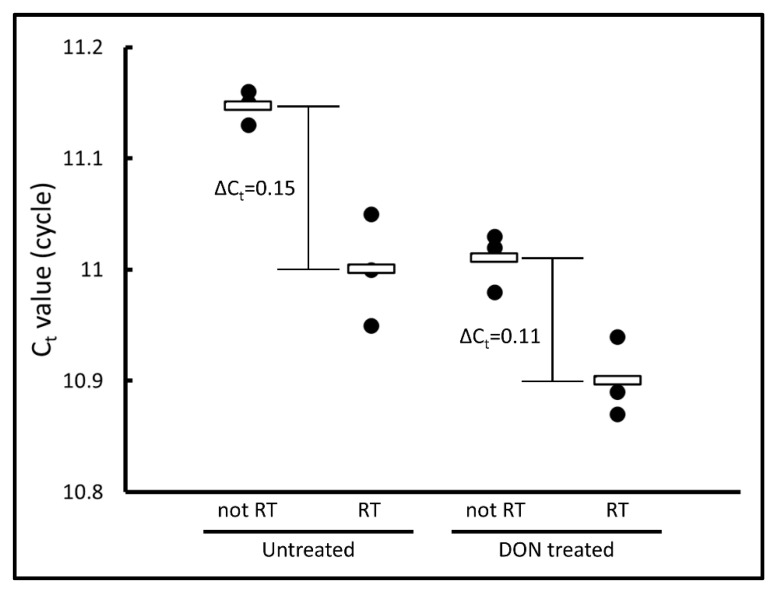
Effect of 2.5 μg/mL DON treatment on transcription. Comparisons of Ct values of reverse-transcribed (RT) sample and not reverse-transcribed (not RT) sample. The “RT” samples contained unremoved plasmid DNA and cDNA. The “not RT” samples did not contain cDNA.

This protein synthesis system has a transcription step using T7 RNA polymerase. We examined the effect on the transcription of the β-galactosidase gene under 2.5 µg/mL DON. Due to the high concentration of the plasmid in the reaction mixture, the elimination of DNA could not be achieved. However, a difference in the amount of DNA was detected between “RT” and “not RT” conditions (untreated conditions in Figure 4). In the presence of 2.5 µg/mL DON, protein synthesis was almost completely inhibited (Figure 3), while transcription was observed (Figure 4). These data indicate that translation, but not the transcription, was inhibited by DON in the system.

## 3. Discussion

Trichothecenes are a large family of compounds, and, recently, masked trichothecenes, such as DON-3-glucoside, have been reported as new natural derivatives found in plants and cereal grains infected with plant pathogens, including *Fusarium* spp. [26,27,28]. Their toxicity to humans and animals varies, but their remains uncertainty in regard to how toxic or not they are.

Trichothecenes bind to and cleave ribosomal RNA [9,10], which leads to inhibition of protein synthesis. Inhibition is the main effect of trichothecenes [11]. Currently, in vitro protein synthesis kits using human cell components are commercially available. We expected these kits to be suitable for the assessment of protein synthesis inhibition caused by trichothecenes. As shown in this study, a kit clearly showed the inhibitory effect of DON. A microbial detoxified metabolite (DOM-1) did not inhibit protein synthesis. Initially, we used water as a solvent, and acetonitrile was also used as a solvent in the system. Acetonitrile sensitivity is very important since some trichothecenes are not soluble in water and many standard reagents are provided in solutions that contain acetonitrile.

Assay systems for the estimation of trichothecene toxicity are not commercially available. Cell-based assays, which are broadly used, require a long time for cell preparation, pre-culture, and other procedures. In addition, it would be difficult to compare the toxicity of trichothecenes under different conditions and among different cell lines. The assay presented here is an in vitro assay using a commercially available kit rather than cell lines. Moreover, as mentioned above, ACN solution can be used in this assay. Furthermore, this system is based on human protein synthesis. Bogus et al. [14] showed that various mycotoxins, including trichothecenes, exhibited different toxic behavior in mammalian cell lines and insect cell line Sf-9. The toxic effects vary among cell lines and cell types. Therefore, an in vitro assay based on the human protein system may be useful for estimating toxicity against a common pathway in humans.

Finally, we presented a comparison of the three major trichothecenes, showing that HT-2 toxin is the strongest inhibitor among them. There are limitations to these results, however, as the trichothecene concentrations used were not molar concentrations. The molar masses of DON, HT-2 toxin, and T-2 toxin are 296, 424, and 467, respectively. The difference in molecular masses between HT-2 toxin and T-2 toxin is only 42 (about 10% of HT-2 mass), so the difference is not expected to be significant. Since the molar concentrations of DON solutions are higher than those of HT-2 and T-2 toxins, the toxicity of DON is expected to be less than of DON treatment when using the same molar concentrations as those of HT-2 toxin and T-2 toxin. Lautraite et al. showed that the IC_50_ of HT-2 and T-2 toxins on the colony formation of granulo-monocytic progenitors was about ten times smaller than that of DON [29]. In addition, it has been shown that T-2 toxin has an IC_50_ that is approximately 300 lower than that of DON on the proliferation of human lymphocytes treated with mitogens [30]. As shown by Sugiyama, HT-2 toxin inhibited LPS-induced NF-κB activity in macrophage-like THP-1 cells at lower doses than T-2 toxin and DON [31]. The findings of this study are consistent with those of studies using human cells in vitro.

## 4. Conclusions

In summary, the assay examined in this study has the advantages of a short operation time (in one day), ease of use, and data stability, because it is a non-cell-based assay whose components are commercially available. Furthermore, acetonitrile, which is frequently used as an organic solvent, can be used for the assay. It is expected that this assay will contribute to the evaluation of the toxicity of a vast number of trichothecenes.

## 5. Materials and Methods

### 5.1. Reagents

Deoxynivalenol (DON) and HT-2 toxin were purchased from Merck (Darmstadt, German). T-2 toxin was purchased from Romer Labs (Getzersdorf, Austria). Deepoxy-deoxynivalenol (DOM-1) was purchased from Toronto Research (Toronto, ON, Canada). The stock solutions were prepared in ultra-pure water or acetonitrile at 4 mg/mL. Before use, each stock solution was diluted to the desired concentration with the respective solvent.

### 5.2. Cell-Free In Vitro Transcription and Translation

Human Cell-Free Protein Expression System (TaKaRa Bio Inc., Shiga, Japan) was used for in vitro transcription and translation. The procedure for the preparation of the mixture and the translation and transcription were conducted according to the instructions with several modifications as shown below. Briefly, 9 µL of cell lysate, 6 µL of Mixture-1, and 1 µL of Mixture-2 in the system were mixed with 0.5 µL of a solution containing a trichothecene at various concentrations or solvent. After incubation for 10 min at room temperature, 2 µL of Mixture-3, 1 µL of T7 RNA polymerase solution (200 U/µL), and 0.5 µL of a plasmid (0.3 μg/mL) containing β-galactosidase gene in the system were added, followed by incubation at 32 °C for 1, 2, or 3 h.

### 5.3. β-Galactosidase Assay

Z buffer (60 mM Na_2_HPO_4_, 40 mM NaH_2_PO_4_, 10 mM KCl, 1 mM MgSO_4_, 50 mM β-mercaptoethanol, pH 7.0) was used as β-galactosidase assay buffer. In total, 4 mg/mL *O*-nitrophenyl-β-D-galactopyranoside (ONPG) and 1 M Na_2_CO_3_ solutions were used as substrate and stop solution, respectively. The reactions were performed in 0.1 mL of Z buffer. For each reaction, 2 µL of the transcribed sample described above and 20 µL of ONPG solution were used. After incubation for 3 min, the reaction was stopped by adding a 50 µL stop solution. The absorbance at 405 nm was determined by a microplate reader (GENios Pro, Tecan, Mannedorf, Switzerland).

### 5.4. RNA and cDNA Preparation and qPCR

The reactions shown in Section 5.2 were performed with or without 2.5 µg/mL DON dissolved in ACN for 2 h followed by RNA preparation and reverse transcription using SuperPrep II Cell Lysis &RT Kit for qPCR (Toyobo Co., Ltd., Osaka, Japan). The preparation was carried out according to the manufacturer’s instructions but with some modifications. Briefly, instead of cell lysate, 20 µL of the reaction mixture was mixed with 25 µL of Lysis Solution with gDNA Remover. In the reverse transcription step of the procedure, we used not only 5×RT Master Mix but also 5×RT Master Mix no-RT Control. Then, 0.2 µL of the lysate sample was subjected to qPCR using THUNDERBIRD SYBR qPCR Mix and a primer set (5′-ATAAACCGACTACACAAATCAG-3′ and 5′-CATAAAGAAACTGTTACCCGTA-3′) for the β-galactosidase gene.

### 5.5. Statistics

FreeJSTAT for Windows 22.0E was used for statistical analysis. Comparisons of relative β-galactosidase activity shown in Figure 2 were analyzed for statistical significance using a two-tailed Student’s *t*-test. Values for the ACN groups were expressed relative to each water group. Multiple comparisons of relative β-galactosidase activity treated with each trichothecene shown in Figure 1, Figure 2 and Figure 3 were analyzed for statistical significance using one-way analysis of variance followed by Dunnett’s test for post hoc analysis. Values for the trichothecene-treated groups were expressed relative to the non-treated group. We considered a significant difference to exist when the significance level was less than 0.01. 

## Data Availability

The data that support the findings of this study are available from the corresponding author upon reasonable request.
